# A Rare Case of Concomitant Fibrillary Glomerulonephritis and Membranous Nephropathy in a Patient With Chronic Inflammatory Demyelinating Polyneuropathy

**DOI:** 10.7759/cureus.92802

**Published:** 2025-09-20

**Authors:** Sheikh Raza Shahzad, Mohammad Hamza, Llewellyn Foulke, Swati Mehta, Krishnakumar Hongalgi

**Affiliations:** 1 Division of Nephrology and Hypertension, West Virginia University School of Medicine, Morgantown, USA; 2 Division of Internal Medicine, Guthrie Cortland Medical Center, Cortland, USA; 3 Division of Pathology, Albany Medical Center, Albany, USA; 4 Division of Nephrology and Hypertension, Albany Medical Center, Albany, USA

**Keywords:** chronic inflammatory demyelinating polyneuropathy, dual glomerulopathy, fibrillary glomerulonephritis, membranous nephropathy, nephrotic syndrome, phospholipase a2 receptor, renal biopsy, subepithelial deposits

## Abstract

Fibrillary glomerulonephritis (FGN) is a rare glomerular disorder characterized by non-branching fibrillary deposits visible on electron microscopy (EM). Rarely, FGN may present with light microscopy (LM) features resembling membranous nephropathy (MN). Immunofluorescence (IF) and EM can distinguish the two entities. We describe, to our knowledge, the first reported case of dual FGN and MN in association with chronic inflammatory demyelinating polyneuropathy (CIDP).

A 68-year-old woman presented with nephrotic syndrome and CIDP. LM demonstrated diffuse mild mesangial expansion. EM revealed mild glomerular basement membrane thickening, subepithelial granular deposits, and diffuse (>90%) podocyte foot process effacement. Additional mesangial and paramesangial intramembranous fibrillary deposits (12-28 nm diameter) were identified. IF demonstrated diffuse granular capillary and mesangial staining for IgG (3+), C3 (2+), IgA (1+), C1q (trace), kappa (4+), and lambda (4+). M-type phospholipase A2 receptor (PLA2R) staining was strongly positive (3+) with a predominantly subepithelial pattern. The patient was treated with furosemide, lisinopril, prednisone, and tacrolimus, resulting in improved proteinuria and edema. She also received intravenous immunoglobulin for CIDP.

FGN may rarely have subepithelial immune deposits resembling MN, but strong anti-PLA2R positivity is unusual and supports the coexistence of two distinct glomerulopathies. The concurrent CIDP raises the possibility of shared immune dysregulation contributing to both renal and neurologic disease. Recognition of such dual pathology is essential, as management may require addressing each condition individually. This case highlights the rare coexistence of FGN and PLA2R-positive MN in a patient with CIDP. Accurate diagnosis requires careful correlation of LM, IF, and EM findings. Further research is warranted to clarify the pathogenesis, clinical implications, and optimal management of dual glomerulopathies.

## Introduction

Fibrillary glomerulonephritis (FGN) is a rare glomerular disease characterized by non-amyloid, non-branching, randomly arranged fibrillary deposits measuring 12-20 nm in diameter [1,2]. Together, FGN and immunotactoid glomerulopathy account for approximately 0.5%-1.4% of native kidney biopsies performed in the United States. FGN carries a poor prognosis, with no established curative therapy other than renal transplantation, which is associated with recurrence in roughly one-third of cases [3]. Chronic inflammatory demyelinating polyneuropathy (CIDP) is an autoimmune demyelinating polyneuropathy, often mediated by autoantibodies against peripheral nerve myelin or nodal/paranodal proteins. Membranous nephropathy (MN) is an immune-mediated glomerular disease characterized by subepithelial immune complex deposition along the glomerular basement membrane, leading to nephrotic syndrome. Accurate diagnosis is challenging, as FGN may be mistaken for MN on light microscopy. Some cases of treatment-refractory MN may, in fact, represent unrecognized FGN [4]. Definitive diagnosis requires electron microscopy (EM). The recent identification of a disease-specific biomarker, DnaJ heat shock protein family (Hsp40) member B9 (DNAJB9), has improved the diagnostic accuracy of FGN [5]. Therapeutic options remain limited. Renin-angiotensin system blockade and various immunosuppressive regimens have been employed with variable success [6]. Rituximab has shown promise in small series, but large clinical trials confirming its efficacy are lacking [7]. While most cases are idiopathic, FGN may be associated with malignancy, monoclonal gammopathy, chronic infection, or autoimmune disease [8]. A literature review revealed only one prior report of FGN occurring with CIDP, in that case associated with monoclonal gammopathy of undetermined significance (MGUS) [1]. To our knowledge, this is the first reported case of FGN in association with CIDP and coincident primary MN.

This article was previously presented as a meeting abstract at the 2019 American Society of Nephrology (ASN) Kidney Week on November 07, 2019.

## Case presentation

A 68-year-old woman presented with a three-month history of bilateral lower extremity weakness and pitting edema, without antecedent illness. Her medical history included hypertension, poorly controlled type 2 diabetes mellitus with peripheral neuropathy, latent tuberculosis (treated with a nine-month course of isoniazid), hyperlipidemia, and hypothyroidism. She denied dyspnea, orthopnea, paroxysmal nocturnal dyspnea, or nonsteroidal anti-inflammatory drug use. Home medications included insulin glargine, insulin aspart, atorvastatin, and lisinopril. Family history was unavailable. She was a former smoker of two large hand-rolled cigarettes daily for 20 years, quitting several years prior. Vital signs were normal except for the blood pressure of 149/81 mmHg. Physical examination revealed 2+ pitting edema and 3/5 strength in proximal and distal lower extremities bilaterally. Tone was normal, with symmetric hyporeflexia (1+) in the lower extremities and baseline numbness/tingling. The Babinski sign was negative. There was no jugular venous distension or lymphadenopathy; the remainder of the examination was unremarkable.

Pertinent labs are summarized in Table 1. Liver function tests were normal. Urinalysis revealed 3+ protein without hematuria or casts. Serologies, including anti-dsDNA antibodies, SPEP/UPEP with immunofixation, antiphospholipid antibodies, ANCA panel, cryoglobulins, HIV, hepatitis panel, and syphilis testing, were negative or normal. CT imaging revealed acute left renal vein and gonadal vein thrombosis; thrombophilia workup was negative. Renal ultrasound showed normal-sized kidneys. The transthoracic echocardiogram was normal.

**Table 1:** Pertinent lab reports of the patient

Test	Result	Normal Range	Interpretation
Hemoglobin A1c	14.50%	4.8 – 5.6 %	High
Serum albumin	1.8 g/dL	2.9 – 4.4 g/dL	Low
pro-BNP	64 pg/mL	< 300 pg/mL	Normal
Free thyroxine (T4)	0.9 ng/dL	0.8 – 1.8 ng/dL	Normal
Thyroid-stimulating hormone (TSH)	6.4 µIU/mL	0.5 – 5.0 µIU/mL	High
Urinalysis	3+ protein	Negative–trace	Proteinuria
24-hour urine protein	6.8 g/day	< 150 mg/day	Nephrotic range proteinuria
Serum creatinine	0.6 mg/dL	0.6 – 1.3 mg/dL	Normal
ANA	0.263889	< 1:40	Positive, high titer
C3	202.4 mg/dL	88 – 201 mg/dL	Mildly elevated
C4	74.7 mg/dL	15 – 45 mg/dL	Elevated
Kappa/Lambda ratio	1.86	0.26 – 1.65	Mildly elevated
Erythrocyte sedimentation rate (ESR)	117 mm/h	< 20 mm/h	Elevated

**Figure 1:**
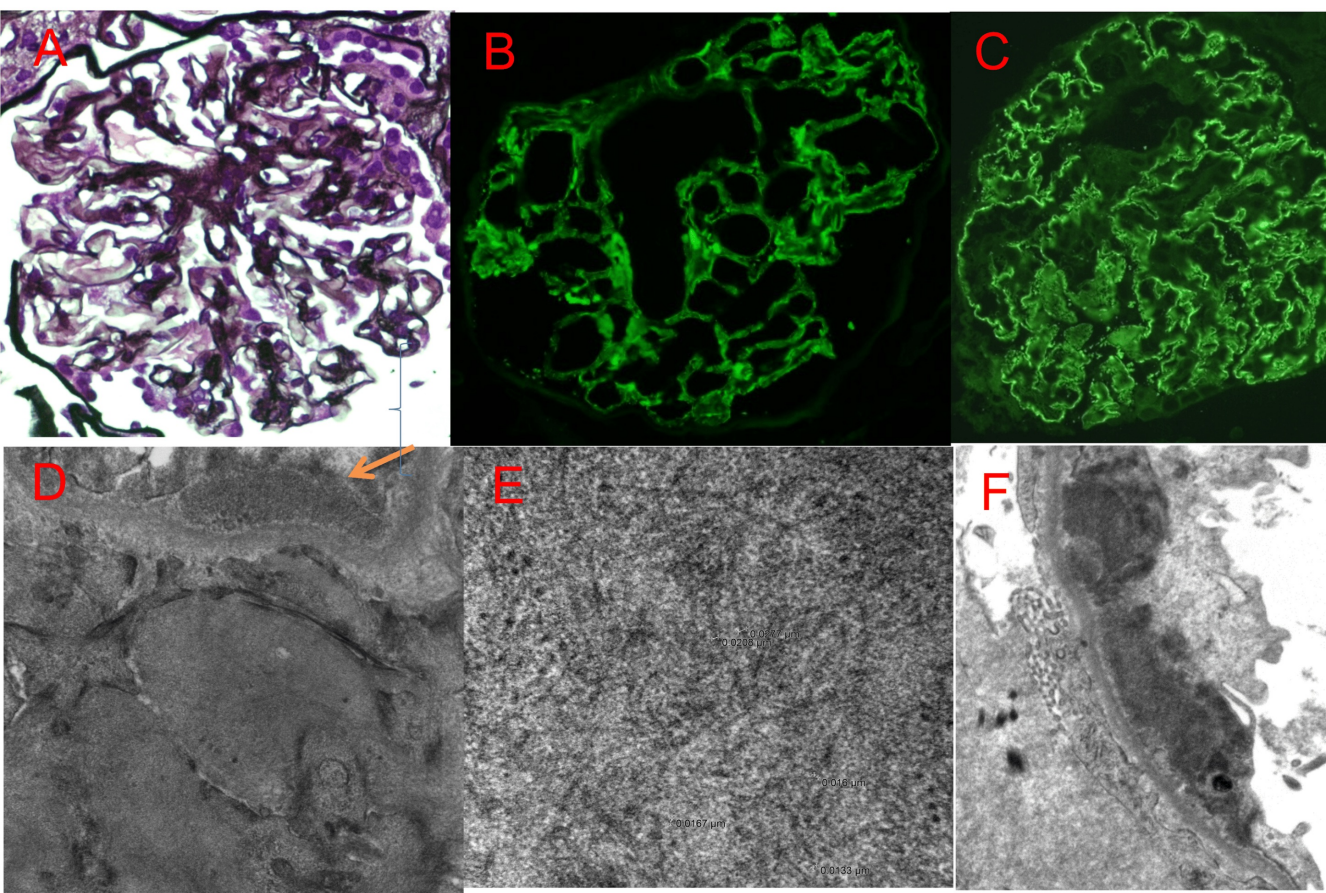
A: Segmental mild mesangial expansion (Jones stain, 600x); B: Direct immunofluorescence for IgG (600x) with global glomerular granular capillary and segmental mesangial staining of 3+ intensity; C: Immunofluorescence for M-type PLA2R (600x) on paraffin-embedded material with global 3+ granular capillary staining. D. Ultrastructural assessment (25,000x) with fibrillary mesangial and adjacent granular epimembranous (arrow) deposits; E. Fibrillary deposits at high magnification (100,000x) with non-branching, randomly arranged 12-28 nm solid fibrils; F. Subepithelial granular electron-dense deposits along peripheral capillary loop with overlying foot process effacement and without neobasement membrane formation, resembling early-stage membranous glomerulonephropathy (10,000X) PLA2R: phospholipase A2 receptor

Light microscopy demonstrated diffuse mild mesangial expansion (Figure 1A), negative Congo red staining, and no Kimmelstiel-Wilson nodules. Immunofluorescence (IF) showed diffuse granular capillary and mesangial staining for IgG (3+) (Figure 1B), C3 (2+), IgA (1+), C1q (trace), kappa (4+), and lambda (4+). IF for M-type phospholipase A2 receptor (PLA2R) was strongly positive (3+) in a subepithelial distribution (Figure 1C). EM revealed mild basement membrane thickening, subepithelial granular deposits, and diffuse (>90%) podocyte foot process effacement (Figure 1F). Additionally, mesangial and paramesangial intramembranous non-branching fibrillary deposits measuring 12-28 nm were identified (Figures 1D, 1E). DNAJB9 staining was negative.

Nerve conduction studies and electromyography showed mixed axonal polyneuropathy, consistent with CIDP. MRI of the spine showed chronic compression deformities of L1 and L4 without acute abnormality. Lumbar puncture revealed 3 WBCs/μL (96% lymphocytes), normal glucose, and normal protein (38 mg/dL). Cerebrospinal fluid testing for infectious and paraneoplastic etiologies was negative. Age-appropriate cancer screening was unremarkable.

The differential diagnosis for nephrotic syndrome included diabetic glomerulopathy, minimal change disease, focal segmental glomerulosclerosis (FSGS), primary membranous nephropathy, and monoclonal gammopathy of renal significance. Lupus nephritis was considered unlikely given negative anti-dsDNA antibodies and normal/elevated complement levels. Renal vein thrombosis was atypical for diabetic nephropathy. While diabetic fibrillosis was considered, the ultrastructural features, like basement membrane involvement, fibril size, and IF profile, favored FGN. The association of CIDP and nephrotic syndrome also raised suspicion for MN or FSGS.

Renal biopsy confirmed primary MN via positive anti-PLA2R staining and extensive podocyte foot process effacement. Unexpectedly, EM also demonstrated fibrillary deposits consistent with FGN, establishing a dual glomerulopathy. Amyloidosis was excluded by Congo red negativity and fibril size. Immunotactoid glomerulopathy was ruled out based on fibril morphology. The patient received heparin and later transitioned to warfarin for renal and gonadal vein thrombosis. Nephrotic syndrome was managed with lisinopril (10 mg twice daily), furosemide (20 mg daily), prednisone (60 mg daily for six weeks), and tacrolimus (1 mg twice daily). For CIDP, she completed five cycles of intravenous immunoglobulin (2.5 g/day). Rituximab was avoided due to a history of latent tuberculosis. Following therapy, she experienced improvement in lower extremity weakness, resolution of edema, and reduction in proteinuria. Over several years of follow-up, her renal function declined but stabilized at a creatinine of 1.92 mg/dL and an estimated glomerular filtration rate of 26 mL/min/1.73 m² (chronic kidney disease (CKD) stage 4).

## Discussion

FGN and immunotactoid glomerulopathy are the most common non-amyloid fibrillary glomerular deposition diseases. Although their relationship remains debated, they exhibit distinct pathological features. FGN is characterized by randomly arranged, non-branching fibrillary deposits measuring 12-20 nm in diameter, whereas immunotactoid glomerulopathy features larger microtubular deposits, typically greater than 30 nm, often arranged in parallel or stacked arrays [9].

While DNAJB9 staining has demonstrated 98% sensitivity and 99% specificity for FGN diagnosis, rare cases with negative staining have been reported. Nasr et al. described two such cases [5]. Historically considered idiopathic, approximately 30%-50% of FGN cases are now known to be associated with malignancy, MGUS, hepatitis C infection, or autoimmune diseases [10]. FGN associated with CIDP is exceedingly rare. The likely pathogenesis involves autoimmune-mediated immunoglobulin deposition within the glomeruli.

Our case is unique due to the coexistence of FGN with primary MN, demonstrated by over 50% podocyte foot process effacement and positive anti-PLA2R staining. Optimal treatment strategies for FGN remain undefined, as no randomized controlled trials have established effective therapies. The prognosis is generally poor. A case series of 27 patients followed over an average of four to five years reported complete remission in 2%, partial remission in 5%, progressive renal disease in 14%, and end-stage renal disease (ESRD) in 45% [6]. Management focuses on treating underlying causes, controlling proteinuria, managing hypertension, and slowing kidney disease progression. To date, immunosuppressive therapies have not demonstrated a clear benefit.

Although there is no single proven pathway, plausible immunologic links tie CIDP, MN, and FGN together, mainly humoral (B-cell/autoantibody) dysregulation, IgG4-related mechanisms (for some cases), shared HLA/immunogenetic susceptibility, and chronic antigenic stimulation. CIDP (and related nodopathies) is antibody-mediated in many patients (e.g., antibodies to contactin-1 and neurofascin), and MN is classically an antibody-mediated disease. This sets up a mechanistic rationale for a single patient to manifest autoantibody-driven disease in the nerve and kidney. FGN can co-exist with other autoimmune disorders, monoclonal gammopathies, or chronic immune stimulation, offering a biological setting in which a patient with CIDP might also develop FGN [1].

## Conclusions

Although extremely rare, primary MN and FGN can present as dual glomerulopathy. Treatment of refractive MN should be evaluated for underlying FGN, as FGN can be mistaken for MN on light microscopy. On extensive review of the literature this, to the best of our knowledge, is a second case of FGN associated with CIDP. More studies are needed to help understand these complex associations. FGN portends a poor prognosis despite immunosuppressive therapy with rapid progression of renal decline, especially in the first year from diagnosis. Although DNJB9 is highly sensitive and specific for FGN, it can be negative for FGN in very rare cases. Although a unifying causal pathway has not been proven, the concurrence of CIDP with MN and FGN may reflect an underlying humoral immune dysregulation, in particular B-cell/autoantibody responses (including IgG4-predominant subsets and anti-paranodal antibodies), together with genetic predisposition and chronic antigenic stimulation that permit immune complex formation/deposition in the glomerulus.
